# Ultrafast 30‐min infusion of a rituximab biosimilar (Truxima)

**DOI:** 10.1002/jha2.812

**Published:** 2023-10-17

**Authors:** Ernesto Pérez‐Persona, Laida Cuevas Palomares, Ariane Unamunzaga Cilaurren, Xabier Gutiérrez López de Ocáriz, Buenaventura Buendía Ureña, Ana Vega González de Viñaspre, Begoña Benito Ibarrondo, María Carmen Molinillo Fernández, Ana Cordero Osúa, Beatriz Benítez Delgado, Juan José García Albás, Miguel Ángel Andrés Moralejo, José María Guinea de Castro

**Affiliations:** ^1^ Department of Haematology Complejo Hospitalario de Navarra Pamplona Spain; ^2^ Bioaraba (Onco‐Haematology Group) Vitoria‐Gasteiz Spain; ^3^ Osakidetza (OSI Araba) Hospital Universitario de Álava (Department of Haematology) Vitoria‐Gasteiz Spain; ^4^ Osakidetza (OSI Araba) Hospital Universitario de Álava (Day‐care Unit) Vitoria‐Gasteiz Spain; ^5^ Osakidetza (OSI Araba) Hospital Universitario de Álava (Department of Pharmacy) Vitoria‐Gasteiz Spain

**Keywords:** rituximab, ultrafast infusion, cost‐efective

## Abstract

In this clinical trial, we demonstrate that ultrarapid fast infusion of rituximab (Truxima) in 30 min with oral premedication is feasible and secure for patients, and reduce the day‐care hospital stays.

1

Rituximab (anti‐CD20) has a well‐documented safety profile. The most common adverse effect is infusion‐related reactions (IRRs). To minimize side effects, the infusion is progressively administered, with a maximum infusion rate of 400 mg/h [1], over 3–4 h. This represents a substantial workload for day‐hospital nursing units. Faster treatment regimens (off‐label) have been explored, with infusion times between 60 and 90 min [2, 3, 4, 5, 6, 7]. Given the challenges posed by the COVID‐19 pandemic to healthcare systems globally, reducing the length of stay in day‐hospital units has become of interest to nursing staff and patients.

We have identified two options to improve the efficiency of day care units. First, there is no established tolerated maximum infusion rate, and a rate of 600 mg/h is well‐established for rheumatoid arthritis [1]. Second, oral premedication may further reduce the infusion time. The aim of this study was to assess the safety of administering a rituximab biosimilar, Truxima, over 30 min with oral premedication. The trial was conducted in accordance with the Declaration of Helsinki and the International Council for Harmonization guidelines for Good Clinical Practice. The institutional review board of the Hospital Universitario de Alava approved the study protocol, and written informed consent was obtained from all patients. Inclusion criteria included patients with lymphoproliferative syndromes (LPS) treated with rituximab (monotherapy or in combination) within the previous 3 months who tolerated 400 mg/. Exclusion criteria were as follows: lymphocyte count > 10 × 10^3^/μL, previous grade II or higher IRRs, and/or severe heart failure (NYHA III‐IV). Rituximab (375 mg/m2) was diluted in a saline solution of 250 mL. Three cohorts were formed, and each cohort was opened if the previous cohort was considered safe, as specified in the protocol (Supplement). Cohort 1: IV premedication for 30 min (5‐mg dexchlorpheniramine and 1‐g paracetamol). The infusion rate was 450 mg/h for 10 min, and 720 mg/h for the remaining 50 min. Cohort 2: premedication as in cohort 1. Rituximab was infused at 450 mg/h for 10 min and at 1800 mg/h for the remaining 20 min. Cohort 3: Oral premedication with 4‐mg dexchlorpheniramine or similar and 1 g oral paracetamol 30–60 min before rituximab. The infusion rate was 450 mg/h for 10 min, and 1800 mg/h for the remaining 20 min. Vital signs were checked before treatment, every 10 min during infusion, and 30 min after infusion. The trial required less than > 2 Grade I‐II IRRs or the absence of Grade II‐IV IRRs to proceed to the subsequent cohort (Supplement). Adverse effects (AEs) and IRRs were assessed independently, using the Common Terminology Criteria for Adverse Events (CTCAE) v 5.0 of the EORTC (November 27, 2017). Clinicaltrials.gov number: NCT05191225. The study conducted between November 2021 and February 2022 included 35 patients (48 infusions). Patient characteristics are summarized in Table 1. One patient received four infusions in different cohorts, one patient received three infusions, and six patients received two infusions. Overall, five Grade I‐II adverse events (10.4%) were reported by an independent observer (IO), and two (4.1%) by a primary investigator (PI). No Grade III‐IV adverse events were observed. The adverse events identified by the IO are described as follows (table 2):

**TABLE 1:** Characteristics of the patients.

Demographic data	*N*: 35
Sex (M/F)	24/11
Age (range), years	63 (36‐91)
Diagnosis	
‐Follicular non‐Hodgkin's lymphoma (NHL)	10
‐Diffuse large B cell lymphoma	10
‐NHL (marginal zone)	8
‐Richter's Syndrome	2
‐Mantle cell lymphoma	1
‐Waldenström´s macroglobulinemia	1
‐Hairy cell leukemia	1
‐Unclassified NHL	2
Number of treatment line	1 (1–2)
Administration	
‐Monotherapy	22 (44%)
‐Combined	26 (56%)
o Combined with:	
▪CHOP	19
▪Bendamustine	5
▪CVP	1
▪GEMOX	1
▪MATRIX	1

Abbreviations: CHOP, cyclophosphamide, adriamicyn, vincristine, prednisone; CVP, cyclophosphamide, adriamicyn, vincristine, prednisone; GEMOX, gemcitabine, oxaliplatin; MATRIX, methotrexate, cytarabine, thiotepa; NHL, non‐hodgkin lymphoma;.

**TABLE 2:** Adverse events and infusion‐related reactions by independent observer.

Infusion number	Cohort	Strategy	IRR by IR*/I (grade)	Symptoms	End at 1800 mL/h	Management
5	1	Mono	I/NC	Desaturation	Yes	No intervention
24	2	R‐CHOP	II/NC	Hypertension	Yes	No intervention
27	2	Mono	I/NC	Hypotension	Yes	No intervention
36	3	Mono	II/II	Flushing, cervical pressure, and nausea	No	Interruption. Methylprednisolone (40 mg)
45	3	Mono	II/II	Pharynx itchy	Yes	Stop temporarily

Abbreviations: IRR IR/I, infusion‐related reaction by independent review/investigator; Mono, rituximab monotherapy; NC, not considered.

Infusion no. 5 (cohort 1): Monotherapy. Grade I IRR: Transient oxygen desaturation to 89% with immediate recovery without additional action. Not considered an IRR by the PI.

Infusion number 24 (cohort 2): Rituximab‐CHOP. Grade II IRR. Transient hypertension was observed, with blood pressure up to 160/86 during the infusion. Spontaneous resolution without additional action. Not considered an IRR by the PI. Infusion number 27 (cohort 2): Rituximab monotherapy. Grade I IRR. Transient hypotension to 97/55 during infusion. No additional medication was administered. Not considered an IRR by the PI.

Infusion n° 36 (cohort 3): Rituximab monotherapy. Grade II IRR consisting of flushing, cervical pressure, and nausea, occurring after 13 min of infusion, 3 min after an infusion rate of 1800 mg/h, without desaturation or changes in blood pressure. The infusion was interrupted, and methylprednisolone (40 mg) was administered. Forty minutes later, all symptoms remitted, and the infusion was restarted at 400 mg/h, ending without further complications. Grade II IRR by PI.

Infusion number 45 (cohort 3): rituximab monotherapy. Grade II IRR. The patient experienced an itchy sensation in the pharynx 4 min after starting the infusion at 450 mg/h, with no changes in vital signs or oxygen desaturation observed. Twenty minutes after the infusion was interrupted, the symptoms disappeared without additional medication. The infusion was restarted at 450 mg/h and increased to 1800 mg/h after 10 min without further complications. Grade II IRR by PI.

Reducing the workload in day‐care hospitals will allow for resource optimization. Several strategies have been employed to decrease the infusion times of anti‐CD20 or anti‐CD38 [2‐4, 7–10). Rituximab (anti‐CD20) is universally used to treat B‐cell LPS. Fast infusion strategies [2, 3, 5‐11], with infusion times between 60 and 90 min, are widely utilized, although 30 min of premedication must be added, resulting in a total minimum infusion time of 90–120 min. The subcutaneous formulation is an alternative with a 5‐min administration [12, 13], although the 2–3‐fold price over the IV formulation leads to a preference for the IV formulation among some healthcare providers. We have demonstrated the safety of a 30‐min infusion, which reduced infusion times by 66%–75% compared to other reported fast infusions and compared favorably with the subcutaneous formulation in terms of efficiency. There were 10.4% Grade I‐II IRRs, and no Grade II‐IV IRRs were independently reviewed. It should be noted that these patients received rituximab in 90‐min infusions. The AEs were mild, and all patients completed the infusion. Only two IRRs recorded by the cohort 3 investigator required intervention. Infusion 36 had common IRR symptoms, requiring corticosteroid therapy and a temporary interruption shortly after increasing the rate to 1800 mg/h. Subsequent infusions were administered at a dose of 400 mg/h. In contrast, the IRRs of Infusion 45 were milder and occurred at 450 mg/h, possibly because the time interval between antihistamine administration and infusion was not sufficiently long. After symptom remission, the patient received an infusion of 1800 mg/h. We recommend an interval of at least 1 h between premedication and treatment administration to ensure proper absorption. Although the toxicity profile seems favorable, the number of patients included in this study was too low to allow for an adequate determination of IRRs. A retrospective study of the administration of rituximab in 30 min has recently been reported, with results that support our clinical trial [14]. These data suggest that ultra‐rapid infusion of rituximab may be feasible, but a larger study is required to confirm this point, as well as to determine an optimal premedication strategy. In conclusion, ultrafast rituximab infusion allows for very short infusion times, significantly reducing the workload in day‐care hospitals and enhancing patient comfort.

## AUTHOR CONTRIBUTIONS

EPP, LCP, and JMGC designed the research protocol. AUC, XGLO, BBU, and AVGV recruit the patients. BBI, MCMF, ACO, and BBD administered the treatment and monitor the patients’ infusions. JJGA and MAAM supervised drug preparation. EPP and LCP wrote de paper. All the authors approved the final version of the paper.

## CONFLICT OF INTEREST STATEMENT

EPP: Presentation: Amgen, Sanofi Kern. Travel and accomodation: Abbvie, Sanofi. XGO: Advisory boards for Janssen, BMS, Sanofi, GSK, Amgen, Astrazeneca, Gilead, JazzPharma. Presentations: Janssen, BMS, Sanofi, GSK, Amgen, SOBI, Abbvie. JMGC: Presentations: Kern. Advisory board for Novartis, Pfizer, and Alexion. LCP, BBU, AUC, AVG, BBI, MCMF, ACO, BBD, JJGA, and MAAM declare not conflict of interest.

## ETHICS STATEMENT

The trial was done in accordance with the Declaration of Helsinki and the International Council for Harmonisation guidelines on Good Clinical Practice. Hospital Universitario de Alava independent ethics committee approved the protocol.

## FUNDING INFORMATION

No funding

## CLINICAL TRIAL REGISTRATION

NCT05191225

## PATIENT CONSENT STATEMENT

All patients provided written informed consent.

## Supporting information

Supporting Information

## Data Availability

Sharing the individual participant data that underlie the results reported in this article (after de‐identification), including the study protocol is allowed. To gain access, data requestors should submit a request to Ernesto.perezpersona@gmail.com
