# Supplementary material for: Ultrafast 30‐min infusion of a rituximab biosimilar (Truxima)
Source: EJHaem. 2023 Oct 17;4(4):1196–9. doi: 10.1002/jha2.812 (PMC10660389; doi:10.1002/jha2.812)
Supplement: Supplementary file 1 — Supporting Information [file JHA2-4-1196-s001.docx]

**Ultrarapid infusion of Truxima in Non-Hodgkin's Lymphoma.**

**The Txagorapid Study. A pilot interventional study**

Principal Investigator

Drs Laida Cuevas Palomares

Ernesto Pérez Persona

Haematology and Haemotherapy Dept.

[1.- GENERAL INFORMATION 3](#_Toc146621108)

[2.- RATIONALE 4](#_Toc146621109)

[2.1 Introduction 4](#_Toc146621110)

[2.2 Study population 8](#_Toc146621111)

[**2.3** References 10](#_Toc146621112)

[3.- AIM AND PURPOSE OF THE STUDY 13](#_Toc146621113)

[3.1. Hypothesis 13](#_Toc146621114)

[3.2 Study population 13](#_Toc146621115)

[3.2.1- PRIMARY ENDPOINT 13](#_Toc146621116)

[3.2.2- SECONDARY ENDPOINTS 14](#_Toc146621117)

[4.- TRIAL DESIGN 15](#_Toc146621118)

[4.1.- Variables 15](#_Toc146621119)

[4.2.- Study description 16](#_Toc146621120)

[4.3.- Trial treatment, dose and treatment regimen 16](#_Toc146621121)

[4.4.- Study duration 16](#_Toc146621122)

[4.5.- Biases and limitations of the study 17](#_Toc146621124)

[5.- SUBJECT SCREENING AND WITHDRAWAL 17](#_Toc146621125)

[6.- SUBJECT TREATMENT 18](#_Toc146621126)

[7.- SAFETY ASSESSMENT 22](#_Toc146621127)

[8.- STATISTICS 27](#_Toc146621128)

[8.1.- Sample Size 27](#_Toc146621129)

[8.2.- Statistical analysis 27](#_Toc146621130)

[9.- DIRECT ACCESS TO SOURCE DATA/DOCUMENTS 28](#_Toc146621131)

[10.- ETHICS 28](#_Toc146621132)

[11.- FUNDING AND INSURANCE 29](#_Toc146621133)

[12.- PUBLICATION POLICY 29](#_Toc146621134)

# 1.- GENERAL INFORMATION

TITLE: Ultrarapid infusion of Truxima in Non-Hodgkin's Lymphoma. The Txagorapid Study. A pilot interventional study.

EudraCT No.: 2019-001750-26

CODE: TxagoRapid.

SPONSOR/PRINCIPAL INVESTIGATOR’S NAME AND ADDRESS: Drs Laida Cuevas Palomares and Ernesto Pérez Persona. Araba University Hospital. Sección Txagorritxu. C/ José Achótegui s/n. 01009. Vitoria-Gasteiz.

MONITOR’S NAME AND ADDRESS: Inés Pérez Francisco. Bioaraba. C/José Atxotegui, s/n, 01009-Vitoria-Gasteiz (Álava). The monitor belongs to the Bioaraba Healthcare Research Institute and is independent of the research team from Osakidetza.

# 2.- RATIONALE

## 2.1 Introduction

Non-Hodgkin's Lymphomas (NHLs) are a heterogeneous group of lymphomas with different behaviours and prognoses. Lymphoma originates when healthy B cells, T cells or NK cells from the lymphatic system change and begin to grow without control, which can lead to tumour formation. As lymphatic tissue is present in essentially all parts of the body, NHL may appear in any of them and disseminate, or metastasise, to almost any organ. It often starts in the lymph nodes, liver, spleen or bone marrow. However, it can also affect the stomach, intestines, skin, thyroid gland, brain or any other part of the body^1^.

Depending on their behaviour, they can be divided into two groups: indolent, or slow-growing, lymphomas and aggressive, or fast-growing, lymphomas. The former are difficult to cure if they spread throughout the body and advance slowly (they can even remain unchanged for many years without needing to be treated). The latter, in contrast, can often be cured even though they may be widespread, although they endanger the patient’s life in the short term if not treated early enough. Depending on the lymphocyte concerned, lymphomas are considered to be B-type (the most common), T-type or NK-type, etc.^2^.

This year, it is calculated that 72,240 new cases of NLH will be diagnosed in the USA (40,080 men and 32,160 women). Although some sub-types of NLH are common in children, NHL is more common in adults, with the risk increasing with age. Indeed, around half of patients are aged 66 years or higher. NLH is the seventh most common cancer in both men and women, and this disease represents 4% of all cancers in the USA^1^. According to data from GLOBOCAN 2012 presented by the Spanish Society of Medical Oncology (SEOM), a five-year prevalence of tumours in both sexes of 6285 cases is estimated for Spain in 2017 (general population)^3^.

There are currently different types of treatment for NLH, including chemotherapy, radiotherapy, targeted therapy with newer drugs that block certain functions of the lymphoma cell and immunotherapy.

The incorporation of immunotherapy in malignant haemopathies over the past 10 years has resulted in a favourable change in the therapeutic options available for Non-Hodgkin's Lymphoma (NHL). Rituximab (anti-CD20) has recently become the cornerstone for the treatment of both aggressive and indolent lymphomas.

Rituximab incorporates the first generation of anti-CD20 monoclonal antibodies to be used as biological therapy in B-type lymphoproliferative syndromes, autoimmune diseases and other conditions in which B lymphocytes proliferate. It is a chimeric murine/human monoclonal antibody obtained by genetic engineering comprising a glycosylated immunoglobulin with the constant regions from human immunoglobulin G1 (IgG1) and murine light- and heavy-chain variable region sequences. Rituximab binds specifically to the membrane antigen CD20, a non-glycosylated phosphoprotein located in pre-B and mature B lymphocytes in both normal and malignant cells^4^.

At a dose of 375 mg/m^2^, rituximab has shown its efficacy and low toxicity profile, and is the most widely used drug in mono- or combination therapy for the treatment of various CD20-positive conditions^5-11^. The therapeutic indications currently approved in the summary of product characteristics for NHL include:

Combination with chemotherapy in the treatment of patients with stage III-IV follicular NHL who have not been treated previously.

The maintenance treatment of patients with relapsed or refractory follicular NLH who respond to induction therapy with chemotherapy alone or in combination with rituximab.

As monotherapy in the treatment of patients with stage III-IV follicular NHL resistant to chemotherapy or who are in their first or second relapse.

In combination with CHOP (cyclophosphamide, doxorubicin, vincristine, prednisolone) chemotherapy for the treatment of patients with CD20-positive diffuse large B-cell NHL (DLBCL).

As indicated in the summary of product characteristics, one notable and characteristic side-effect is hypersensitivity reactions (severe dyspnoea, bronchospasm or hypoxia). The causes of this type of reaction are the murine nature of the drug, tumour lysis syndrome and cytokine release syndrome^12^, which occur upon administration. The prevention of such hypersensitivity reactions requires slow administration of the drug. The first administration is the slowest, with an initial infusion rate of 50 mg/h in the first 30 minutes. Subsequently, and in the absence of a reaction, the rate can be increased on steps of 50 mg/h every 30 minutes up to a maximum of 400 mg/h. Subsequent infusions can start at a rate of 100 mg/h, increasing in steps of 100 mg/h every 30 minutes up to a maximum of 400 mg/h. As such, each infusion of rituximab, together with the premedication administered to prevent an infusion-related reaction, can take more than 3 hours, thus representing a significant workload for the day hospital and obliging patients to remain at the hospital for several hours.

To overcome these drawbacks, on 02 June 2017, the FDA approved a new formulation of [rituximab](https://www.cancer.gov/Common/PopUps/popDefinition.aspx?id=CDR0000045525&version=Patient&language=Spanish) in combination with the [enzyme](https://www.cancer.gov/Common/PopUps/popDefinition.aspx?id=CDR0000046081&version=Patient&language=Spanish) hyaluronidase, which allows subcutaneous administration in 5 or 7 minutes instead of intravenous infusion.

The clinical experience with the subcutaneous MabThera formulation in Non-Hodgkin's Lymphoma is based on data from a phase III (SABRINA BO22334), randomised multi-centre non-inferiority clinical trial in untreated patients with follicular lymphoma in which, in addition to investigating the non-inferiority as primary endpoint, the pharmacokinetic profile, efficacy and safety of the subcutaneous MabThera formulation (combination of CHOP or CVP with rituximab SC vs IV) were also investigated, demonstrating the same efficacy and safety thereof^12^.

The approval stipulates that the [subcutaneous](https://www.cancer.gov/Common/PopUps/popDefinition.aspx?id=CDR0000045914&version=Patient&language=Spanish) treatment can only be used after patients have received at least one treatment of IV rituximab with good tolerance due to the risk of hypersensitivity and other serious side-effects during the first infusion^13^. The subcutaneous administration of rituximab has allowed the workload at day hospitals to be reduced and improved patients’ quality of life.

In July 2017, Kern Pharma launched Truxima, the first biosimilar for rituximab. Pharmacokinetics and pharmacodynamics studies comparing Truxima with MabThera showed that the former is very similar from a chemical structure, purity and biological activity viewpoint. In real life, Truxima has been compared with MabThera in various phase I/III clinical trials and has been shown to be not inferior in terms of efficacy, and to have similar effects in terms of tolerability and safety^14-16^.

Currently, given the same efficacy and lower cost, Osakidetza has prioritised use of the biosimilar rather than MabThera. The subcutaneous administration of rituximab has been used at the Araba University Hospital since 2017. Given approval of biosimilar rituximab at a lower price, the possibility of returning to intravenous administration has been evaluated. However, as noted above, intravenous administration requires a longer administration time. This, in turn, leads to a higher workload for the day hospital and a longer stay there for patients.

Since 2010, the Haematology Department at the Araba University Hospital has protocolised that those individuals who tolerate the maximum rate of 400 mg/h at the first infusion will receive the second and subsequent infusions at an initial rate of 400 mg/h. No significant complications have been reported since the start of this protocol. Furthermore, the use of this protocol can shorten the infusion time to approximately two hours.

There have been some attempts to shorten the administration time for intravenous rituximab. In this regard, some studies have reported that the administration of rituximab over 60 minutes is viable and risk-free^16,17^. However, even with this infusion rate, the overall administration time (together with the premedication) would not decrease to less than 2 hours.

In order to reduce the administration regimen and provide a genuine alternative to the administration of subcutaneous rituximab, it has been proposed to perform an ultra-rapid infusion on 30 min.

As noted in the summary of product characteristics, the maximum permitted concentration of rituximab is 4 mg/mL. For a normal individual with a body surface area of 1.8 m^2^ (weight of 70 kg and height of 1.7 m), the total dose of rituximab is 675 mg, therefore dilution in 250 ml of saline would lead to a rituximab concentration of 2.7 mg/ml.

The recommended infusion rate from the second administration onwards (provided the maximum rate of 400 mg/h is achieved in the first infusion) is to start at 100 mg/h and progressively increase this to 400 mg/h. As discussed previously, standard practice at our hospital, in accordance with numerous publications, is direct administration at 400 mg/h from the second infusion onwards. Moreover, the summary of product characteristics allows faster infusions in patients with rheumatoid arthritis, who can receive a faster infusion at the second and subsequent sessions, starting at a rate of 250 mg/hour for the first 30 minutes and then 600 mg/hour for the following 90 minutes. If this faster infusion is tolerated, the same regimen can be used when subsequent infusions are administered.

With the experience at our hospital, we have not observed infusion-related complications with the administration of 400 mg/h, and given the indications in the summary of product characteristics the option to increase the infusion rate further still remains. We believe that an increase in the infusion rate should not have an effect in safety, would reduce the workload at the day hospital and would impact patients’ quality of life by reducing their stay at the hospital.

The aim of this pilot study is therefore to evaluate the safety of an ultra-rapid infusion of Truxima in NHL patients over 30 minutes.

## 2.2 Study population

Patients diagnosed with non-Hodgkin's Lymphoma who are receiving rituximab as per standard practice following the indications established by Osakidetza, who attend the Haematology Department at the Araba University Hospital, will be included provided they comply with all the inclusion criteria and none of the exclusion criteria set out in the corresponding section.

- 1. **References**

^1^[https://www.cancer.net/es/tipos-de-c%C3%A1ncer/linfoma-no-hodgkin/subtipos-de-lnh](https://www.cancer.net/es/tipos-de-cáncer/linfoma-no-hodgkin/subtipos-de-lnh)

^2^[https://www.aecc.es/es/todo-sobre-cancer/tipos-cancer/linfoma-no-hodgkin/que-es linfoma-no-hodgkin](https://www.aecc.es/es/todo-sobre-cancer/tipos-cancer/linfoma-no-hodgkin/que-es%20linfoma-no-hodgkin)

^3^<https://seom.org/seomcms/images/stories/recursos/LaCifras_delcanceenEspana2018.pdf>

Dr. Edgardo E. Espinosa EstradaI, Dr. Luis G. Ramón Rodríguez; Dra. Lissete Izquierdo CanoI et al. Rituximab: history, pharmacology and perspectives; Instituto de Hematología e Inmunología. Ciudad de La Habana, Cuba. Revista Cubana de Hematología, Inmunología y Hemoterapia. 2010; 26(1)186-197.

^4^Maloney DG, Grillo-Lopez AJ, White CA. IDEC-C2B8 (Rituximab) anti CD20 monoclonal antibody in patients with relapsed low-grade non- Hodgkin´s lymphoma. Blood 1997;90:2188-95.

^5^Wilson WH. Chemotherapy sensitization by Rituximab: Experimental and clinical evidence. Semin Oncol 2000:6:30-6.

^6^Maloney DG. Preclinical and phase I and II trial of Rituximab. Semin Oncology 1999;5:74-8.

^7^Czuczman MS, Grillo-Lopez AJ, White CA. Treatment of patient with low-grade-B-cell lymphoma with the combination of chimeric anti-CD20 monoclonal antibody and CHOP chemotherapy. J Clin Oncol 1999;17:268-76.

^8^Marcus R, Imrie K, Belch A. An international multicentre, randomized, open-label phase III trial comparing Rituximab added to CVP chemotherapy to CVP chemotherapy alone in untreated stage III/IV follicular non-Hodgkin´s lymphoma: Final analysis. Blood 2003;102:28ª

^9^Salles GA, Seymour JF, Feugier P, et al. Rituximab maintenance for 2 years in patients with untreated high tumor burden follicular lymphoma after response to immunochemotherapy (Abstract 8004) J Clin Oncol. 2010;28(Suppl 15s)

^10^[Davies A](https://www.ncbi.nlm.nih.gov/pubmed/?term=Davies%20A%5bAuthor%5d&cauthor=true&cauthor_uid=28476440), [Merli F](https://www.ncbi.nlm.nih.gov/pubmed/?term=Merli%20F%5bAuthor%5d&cauthor=true&cauthor_uid=28476440), [Mihaljević B](https://www.ncbi.nlm.nih.gov/pubmed/?term=Mihaljević%20B%5bAuthor%5d&cauthor=true&cauthor_uid=28476440), [Mercadal S](https://www.ncbi.nlm.nih.gov/pubmed/?term=Mercadal%20S%5bAuthor%5d&cauthor=true&cauthor_uid=28476440), [Siritanaratkul N](https://www.ncbi.nlm.nih.gov/pubmed/?term=Siritanaratkul%20N%5bAuthor%5d&cauthor=true&cauthor_uid=28476440), [Solal-Céligny P](https://www.ncbi.nlm.nih.gov/pubmed/?term=Solal-Céligny%20P%5bAuthor%5d&cauthor=true&cauthor_uid=28476440) et al. Efficacy and safety of subcutaneous rituximab versus intravenous rituximab for first-line treatment of follicular lymphoma (SABRINA): a randomised, open-label, phase 3 trial. The Lancet, Haematology, May 02, 2017

^11^ Wendy H. Vogel, MSN, FNP, AOCNP® Infusion Reactions: Diagnosis, Assessment, and Management. Clinical Journal of Oncology Nursing . Volume 14, Number 2

^12^ [Kim](https://www.clinicalkey.com/#!/search/Kim%20Won Seog/%7B%22type%22:%22author%22%7D) WS, [Buske C](https://www.clinicalkey.com/#!/search/Buske%20Christian/%7B%22type%22:%22author%22%7D), [Ogura O](https://www.clinicalkey.com/#!/search/Ogura%20Michinori/%7B%22type%22:%22author%22%7D) et al. Efficacy, pharmacokinetics, and safety of the biosimilar CT-P10 compared with rituximab in patients with previously untreated advanced-stage follicular lymphoma: a randomised, double-blind, parallel-group, non-inferiority phase 3 trialLancet Haematology, The, 2017-08-01, Volume 4, Issue 8, Pages e362-e373.

^13^ Won-Seog Kim, MD, PhD^1*^, Christian Buske, MD^2^, Larry W Kwak, MD, PhD^3^, Michinori Ogura, MD, PhD^4^, Bertrand Coiffier, MD, PhD^5^, SangJoon Lee, PhD^6*^ and SuYoung Kim^6^. 1616 Similar Efficacy and Safety of CT-P10 and Reference Rituximab in Patients with Advanced Stage Follicular Lymphoma: Updated Phase III Study Results Program: Oral and Poster Abstracts Session: 623. Mantle Cell, Follicular, and Other Indolent B-Cell Lymphoma—ClinicalStudies. ASH 2018

^14^ Michinori Ogura, Juan Manuel Sancho, Seok-Goo Cho, Hideyuki Nakazawa, el at. 1596 Comparison of Efficacy and Safety of Biosimilar CT-P10 to Rituximab in Patients with Previously Untreated Low Tumor Burden Follicular Lymphoma (LTBFL): A Randomized Phase III Study. Program: Oral and Poster Abstracts. Session: 623. Mantle Cell, Follicular, and Other Indolent B-Cell Lymphoma—Clinical Studies: Poster I. ASH 2018

^15^ [Nagwa Ibrahim](https://journals.sagepub.com/doi/abs/10.1177/1078155208100527), Pharm D, [Ahmed Al Eid](https://journals.sagepub.com/doi/abs/10.1177/1078155208100527), PhD. Rapid Infusion Rituximab Changing Practice for Patient Care.  January 26, 2009 Other. SAGE journals, Vol 15, issue 3.

^16^ [Antonio Salar](https://onlinelibrary.wiley.com/action/doSearch?ContribAuthorStored=Salar,+Antonio), [Dolors Casao](https://onlinelibrary.wiley.com/action/doSearch?ContribAuthorStored=Casao,+Dolors), [Marta Cervera](https://onlinelibrary.wiley.com/action/doSearch?ContribAuthorStored=Cervera,+Marta),et al. Rapid infusion of rituximab with or without steroid‐containing chemotherapy: 1‐yr experience in a single institution. First published: 19 July 2006, European journal of haematology, Vol 77, issue4, pages 338-340

^17^ Lang SPD, Hagger C, Pearson A. Safety of rapid rituximab infusion in adult cancer patients: a systematic review. Int. J. Nursing Practice. 2011; 17: 357-369.

^18^ García-García, J. A., Reding-Bernal, A., & López-Alvarenga, J. C. (2013). Cálculo del tamaño de la muestra en investigación en educación médica. Investigación en educación médica, 2(8), 217-224.

^19^ Babbie, E. (2000). Fundamentos de la investigación social (No. 300.72 B112f). México, MX: International Thomson Ed.

# 3.- AIM AND PURPOSE OF THE STUDY

## 3.1. Hypothesis

Increasing the infusion rate for rituximab is as safe as administration under standard practice (400 mg/h).

## 3.2 Study population

**3.2.1- PRIMARY ENDPOINT**

To evaluate the safety of a rapid, ultrarapid and ultrarapid plus infusion of rituximab based on the percentage of grade 2/3 or higher infusion-related reactions in comparison with the administration of rituximab as per standard clinical practice at the standard rate of 400 mg/h.

Infusion-related reactions are defined as adverse events that occur in the first 24 hours after infusion of rituximab and which are considered to be related to infusion of the drug in the investigator's opinion.

Infusion-related reactions will be graded as the EORTC guidelines: Common Terminology Criteria for Adverse Events (CTCAE) Version 5.0 of EORTC (27 November 2017)

| **GRADE** | **REACTION** |
| --- | --- |
| Grade I: Mild | - - Transitory reaction   - Infusion not suspended   - No indication for intervention |
| Grade II: Moderate | - - Infusion suspended, with rapid recovery of symptoms after symptomatic treatment (e.g., antihistamines, NSAIDs, analgesia, volume support)   - Prophylactic medication for less than 24 hours |
| Grade III: Severe | - - Prolonged symptoms (e.g., slow response to symptomatic medication or suspension of infusion)   - Symptoms recur after initial improvement   - Hospitalisation indicated |
| Grade IV: Very severe | - - Life-threatening symptoms   - Urgent intervention indicated |
| Grade V: | - - Related death |

**3.2.2- SECONDARY ENDPOINTS**

- Determine the intensity of the infusion-related reactions between rapid, ultrarapid and ultrarapid plus infusions of rituximab.
- Determine the time until the infusion-related reaction.

# 4.- TRIAL DESIGN

**4.1.- Variables**

Primary variable:

-Percentage of grade 2/3 or higher infusion-related reactions for the rapid, ultrarapid and ultrarapid plus infusions in comparison with the administration of rituximab as per standard clinical practice at the standard rate of 400 mg/h.

** Infusion-related reactions are defined as adverse events that occur in the first 24 hours after infusion of rituximab and which are considered to be related to infusion of the drug in the investigator's opinion.*

Secondary variables:

- Grade of infusion-related reactions (1-5 as per CTCAE version 4.0) occurring in each study group.

- Time (in minutes) between the start of infusion and onset of the infusion-related reaction.

Other variables:

- Age (years)

- Sex (male/female)

- Infusion group (rapid, ultrarapid or ultrarapid plus)

- Treatment type (induction/maintenance)

- Therapy type (rituximab in monotherapy/combination therapy - specify the type of concomitant treatment: chemotherapy, radiotherapy, targeted therapy, etc.)

- Lymphoma type (specify diagnosis)

- Rituximab cycle number

- Blood pressure, temperature, oxygen saturation and heart rate before starting and after finishing rituximab infusion

**4.2.- Study description**

The intention is to perform a single-centre interventional pilot study to evaluate the safety of administering intravenous rituximab as a rapid, ultrarapid or ultrarapid plus infusion in comparison with standard clinical practice at the standard rate of 400 mg/h.

**4.3.- Trial treatment, dose and treatment regimen**

In the case of patients assigned to the **rapid infusion group**, after the IV administration of premedication (generally dexchlorpheniramine 5 mg IV and paracetamol 1 g IV), the standard dose of rituximab (375 mg/m^2^ diluted in 250 ml of saline) will be administered over one hour, as follows: first 10 minutes at 450 mg/hour and subsequent 50 minutes at 730 mg/hour.

For the **ultrarapid infusion group**, after the administration of premedication (generally dexchlorpheniramine 5 mg IV and paracetamol 1 g IV), the standard dose of rituximab (375 mg/m^2^ diluted in 250 ml of saline) will be administered over half an hour, as follows: first 10 minutes at 450 mg/hour and subsequent 20 minutes at 1800 mg/hour.

And for the **ultrarapid plus infusion group**, after the administration of premedication (dexchlorpheniramine 4 mg orally and paracetamol 1 g orally), the standard dose of rituximab (375 mg/m^2^ diluted in 250 ml of saline) will be administered over half an hour, as follows: first 10 minutes at 450 mg/hour and subsequent 20 minutes at 1800 mg/hour.

**4.4.- Study duration**

12 months.

**4.5.- Biases and limitations of the study**

The pilot study proposed does not have a sufficient sample size to demonstrate statistically significant differences in the percentage of infusion-related reactions that may occur in each study group compared with the percentage of infusion-related reactions that occur in standard treatment with rituximab at the standard rate (i.e., 400 mg/h). However, as the maximum infusion rate described in the scientific literature is 60 min, the present study appears to be the first to evaluate the safety with ultrarapid infusion rates of 30 min. As such, despite lacking sufficient power, it has been decided to perform an initial pilot study to evaluate the safety of ultrarapid infusions before carrying out a subsequent multicentre clinical trial.

Given the escalation nature of the study, patients will not be assigned randomly to the three study groups and, if they do not suffer any adverse infusion-related reaction, the same patient may receive the infusions described for the three study groups. This may bias the results as the characteristics of the patient and the type of lymphoma they suffer may affect the percentage of infusion-related reactions that occur with each infusion type to be studied. However, and despite the small sample size, the intention is to analyse infusion-related reactions based on the characteristics of the disease.

# 5.- SUBJECT SCREENING AND WITHDRAWAL

Inclusion criteria:

- Patients who have tolerated the maximum intravenous administration rate for rituximab (400 mg/h) during the first or second infusion.
- The last infusion of rituximab must have taken place in the previous 3 months.
- All patients receiving rituximab may be included irrespective of whether they do so as monotherapy or in combination with chemotherapy and irrespective of the line in which they are receiving it.
- Patient in both induction and maintenance treatment will be included.
- All patients must sign the informed consent.

Exclusion criteria:

- Patients with an initial absolute lymphocyte count >10x10^3^ cells/µL.
- Patients who have presented hypersensitivity and serious adverse effects (grade 2 or higher) after the first or second infusion.
- Severe heart failure (NYHA class III-IV) or severe uncontrolled heart disease.
- Respiratory failure, uncontrolled COPD/severe asthma.
- Patients allergic to the premedication: paracetamol or polaramine.
- Severe active infection.
- Pregnant patients. *
- Refusal to participate

* For patients of child-bearing age (women of child-bearing age are considered to be those between menarche and post-menopause, except for those who are permanently sterile), a pregnancy test must be performed prior to treatment.

Women of child-bearing age participating in the trial must use effective contraceptive methods during treatment and for up to 12 months afterwards. These methods include:

- - - Sexual abstinence.
    - Female sterilisation.
    - Sterilisation of the (male) partner.
    - Intra-uterine device (IUD)

Withdrawal criteria:

- Onset of one or more grade 2 or higher infusion-related reactions
- The presence of four or more mild to moderate infusion-related reactions (25%) in the 16 patients of the corresponding cohort

# 6.- SUBJECT TREATMENT

When the patient attends the haematology surgery and has tolerated the maximum intravenous administration rate for rituximab (400 mg/h) during the first or second infusion, they will be invited to participate in the study and will be given the informed consent. If the patient complies with all the inclusion criteria and none of the exclusion criteria, and decides to participate, they will be given an appointment to attend the day hospital for administration of their treatment cycle at the infusion rate being studied at that time.

Three successive intervention groups with different infusion rates (rapid, ultrarapid and ultrarapid plus) will be established. The procedure for each cohort will be considered to be safe if fewer than four mild to moderate infusion-related reactions occur in the 16 patients from the corresponding cohort (25% of reactions). The onset of one or more grade 2 or higher infusion-related reactions will imply that the cohort is not safe and the clinical trial will be suspended. Before commencing the next cohort, a haematology specialist not linked to Araba University Hospital Haematology Department will carry out a safety monitoring. This will involve an evaluation of all infusions performed, with access to the progress reports (medical and nursing) to determine whether a reaction has occurred and the grade thereof. In an attempt to minimise the influence of the investigator on the safety evaluation, the safety monitor will receive the progress reports after references to the grade of the infusion-related reaction contained in the electronic medical records have been censored.

Rituximab will be diluted in 250 mL provided the maximum concentration established in the summary of product characteristics (max. 4 mg/mL) is not exceeded. The rituximab dose will not be changed.

If the patient is receiving concomitant chemotherapy, this will be administered after rituximab once the absence of adverse reactions has been confirmed.

The interventions applied in each of the three study groups are described below:

RAPID INFUSION GROUP

Administration of the IV premedication half an hour prior to the infusion of rituximab as per the standard regimen; generally, dexchlorpheniramine 5 mg IV and paracetamol 1 g IV half an hour prior to rituximab.

The rituximab dose will then be administered at the standard dose: 375 mg/m^2^ diluted in 250 mL of saline over one hour, as follows:

- - - First 10 minutes at 450 mg/hour
    - Subsequent 50 minutes at 720 mg/hour

A total of 16 infusions will be included in this group and the safety thereof will be evaluated. If the number of AEs is not higher than normal (more than three infusion-related reactions), the second intervention will commence. In the event that the number of infusion-related reactions is higher than in standard clinical practice, the study will be suspended without continuing with the following groups.

ULTRARAPID INFUSION GROUP

Administration of the IV premedication half an hour prior to the infusion of rituximab as per the standard regimen; generally, dexchlorpheniramine 5 mg IV and paracetamol 1 g IV half an hour prior to rituximab.

The rituximab dose will then be administered at the standard dose: 375 mg/m^2^ diluted in 250 mL of saline over half an hour, as follows:

- - - First 10 minutes at 450 mg/hour.
    - Subsequent 20 minutes at 1800 mg/hour.

A total of 16 infusions will be included in this group and the safety thereof will be evaluated. If the number of infusion-related reactions is not higher than normal (more than three infusion-related reactions), the third intervention will commence. In the event that the number of infusion-related reactions is higher than in standard clinical practice, the study will be suspended without continuing with the following group.

ULTRARAPID PLUS INFUSION GROUP

Administration of the oral premedication half an hour prior to the infusion of rituximab as per the standard regimen for administration of subcutaneous rituximab: dexchlorpheniramine 4 mg orally and paracetamol 1 g orally half an hour prior to rituximab.

The rituximab dose will then be administered at the standard dose: 375 mg/m^2^ diluted in 250 mL of saline over half an hour, as follows:

- - - First 10 minutes at 450 mg/hour.
    - Subsequent 20 minutes at 1800 mg/hour.

A total of 16 infusions will be included in this group and the safety thereof will be evaluated. This ultrarapid infusion regimen will be considered to be safe if the number of infusion-related reactions is three or less.

Safety will be evaluated by monitoring infusion-related reactions, including all haematological and non-haematological serious and non-serious adverse events related to the study drugs. If an infusion-related reaction occurs, the standard administration rate of 400 mg/h will be used.

To that end, vital signs (temperature, blood pressure, heart rate and oxygen saturation) will be monitored every 10 minutes and all events that occur will be recorded in the CRF. At 24 hours post-infusion, the study nurse will contact the patient by telephone to determine whether any adverse reaction has occurred.

The other evaluations to be performed during the study are the same as those in standard clinical practice. Diagnostic tests and the pharmacological treatment will be carried out irrespective of participation of the patient in the study.

Study diagram:


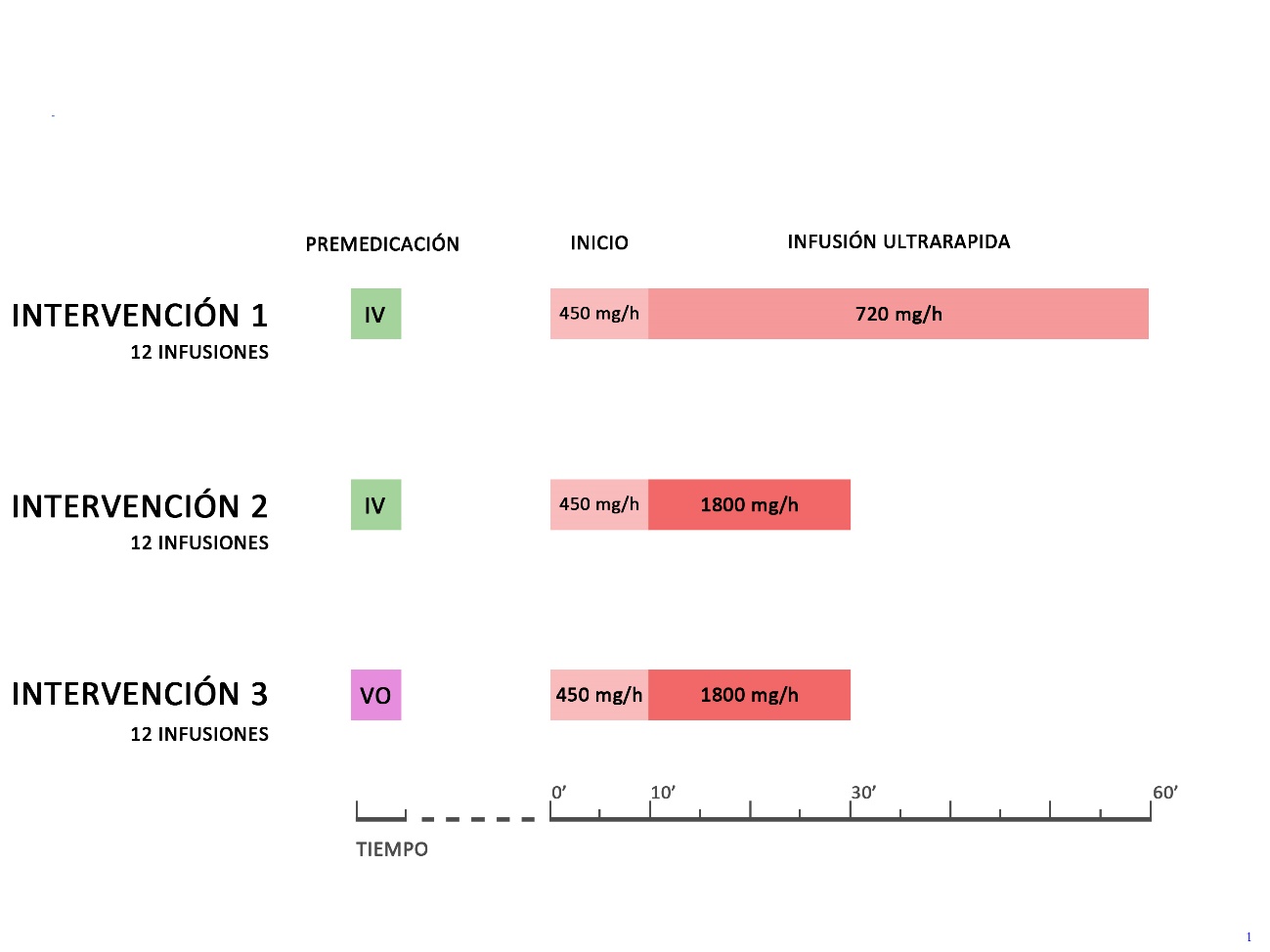


# 7.- SAFETY ASSESSMENT

Safety assessments will be carried out in accordance with Good Clinical Practice guidelines and current legislation.

The sponsor is responsible for managing the adverse events (AEs) recorded during this study. The sponsor is responsible for the expedited reporting of suspected unexpected serious adverse reactions (SUSARs), annual reports or any other relevant safety information to the health authorities (AEMPS, CCAA) and clinical research ethics committees (CRECs).

The sponsor is also responsible for reporting safety information to the investigators.

- Definitions. Classifications.

Adverse event (AE):

Any incident that is harmful to the health of the patient or clinical trial subject treated with a medicinal product, even if it does not necessarily have a causal relationship with said treatment.

An AE may therefore be any unfavourable and unintentional sign (including an abnormal laboratory finding), symptom, or disease temporally associated with the use of an investigational medicinal product, whether or not considered related to the medicinal product.

Adverse Reaction (AR):

Any unintended harmful reaction to an investigational medicinal product, regardless of the dose administered.

In contrast to an AE, in the case of an AR there is a suspected causal relationship between the investigational medicinal product and the adverse event.

All AEs and ARs will be classified as mild, moderate or severe based on their severity.

Severity Criteria

The severity will be defined as specified below:

- Mild: the patient experiences discomfort that does not interfere with their daily activity.
- Moderate: sufficient discomfort to reduce or affect daily activity.
- Severe: inability to work or undertake daily activity.

Causality criteria

The sponsor will classify adverse events based on their causality relationship with the drug, as per the Karsch and Lasagna algorithm (1977), as:

- - - Definitive: there is a reasonable time sequence between administration of the drug and onset of the adverse event. Said event coincides with the adverse reactions described for the drug, improves upon suspension thereof, reappears after re-administration and cannot be explained by other causes.
    - Probable: there is a reasonable time sequence between administration of the drug and onset of the adverse event. Said event coincides with the adverse reactions described for the drug, improves upon suspension of treatment and cannot be explained by other causes.
    - Possible: there is a reasonable time sequence between administration of the drug and onset of the adverse event. Said event coincides with the adverse reactions described for the drug but may be explained by other causes.
    - Conditional or improbable: there is a reasonable time sequence between administration of the drug and onset of the adverse event. Said event does not coincide with the adverse reactions described for the drug and can be explained by other causes.
    - Unrelated: there is no reasonable time sequence between administration of the drug and onset of the adverse event. Said event does not coincide with the adverse reactions described for the drug and can be explained by other causes.

For the purposes of expedited reporting, the categories definitive, probable and possible from the Karsch and Lasagna algorithm (1977) will be considered to be related and the category conditional or improbable will be considered to be unrelated.

The principal investigator at the research site, or a person designated by the PI, will be responsible for determining the possible relationship with the study treatment.

Serious Adverse Event (SAE) and Serious Adverse Reaction (SAR)

Any adverse reaction or adverse event which, at any dose:

- - - Causes the death of the patient.
    - Endangers the patient’s life.
    - Requires hospitalisation or prolongation of hospitalization of the patient.
    - Causes permanent or significant disability or incapacity.
    - Leads to a congenital anomaly/birth defect.

For reporting purposes, those suspected adverse events or adverse reactions considered to be important from a medical point of view will be considered to be serious even if they fail to comply with the above criteria, including important medical events requiring an intervention to prevent any of the consequences described above from occurring. Similarly, all suspected transmissions of an infectious agent via a drug will be reported as serious. The concept “serious”, defined above, should not be confused with “severe”, which refers to the severity of the AE or AR (mild/moderate/severe).

Suspected Unexpected Serious Adverse Reaction (SUSAR)

Any adverse reaction whose nature or severity does not correspond with the reference information for the product (for example, the Investigator's Brochure in the case of an investigational medicinal product that is not authorised for marketing, or the summary of product characteristics for the product if it is an authorised drug).

The unexpected nature of an adverse reaction is based on the fact that it has not been observed previously and will not be based on what might be expected given the pharmacological properties of the drug.

SAEs that do not comply with the SUSAR criteria and which are associated with blinded studied will be reported to the health authorities unblinded.

Management of adverse events/pregnancy

Recording of adverse events/pregnancy

All adverse events/pregnancies notified either spontaneously by the patient or during the interviews held during the study visits will be recorded. AEs/pregnancies will be recorded from the moment the patient signs the informed consent (IC) up until 30 days after administration of the last dose of the investigational medicinal product and/or final visit.

In the event of pregnancy, the patient will be excluded from the study.

Information concerning the AEs that may have occurred in the period of time between the previous visit and the current visit must be requested.

All AEs/pregnancies must be documented in the patient’s medical records and the case report form (CRF).

All AEs must be followed up until they have resolved, or for at least 30 days after withdrawal of the study drugs (whichever occurs first), until the toxicity returns to a grade ≤ 1, or until the toxicity is considered to be irreversible.

Any pregnancy that occurs during the study, and its outcome, must be recorded and followed-up to rule out congenital malformations or birth defects. The following information should be recorded:

- Normal birth, miscarriage or abortion (any congenital abnormality detected in the aborted foetus must be documented), stillbirth, congenital abnormality.
- Neonatal deaths occurring in the first 30 days after birth.
- Death of an infant subsequent to those 30 days if the investigator suspects this to be related to intrauterine exposure to the study drug.
- All infants born after foetal exposure must be followed-up for the first 12 months after birth.

Any exacerbation of a pre-existing disease that occurs subsequent to start of the study treatment is also considered to be an adverse event.

Any abnormal finding in blood tests that the investigator considers to be clinically significant and which requires adjustment of the dose of study drug, temporary or permanent suspension of said treatment or any type of intervention of diagnostic evaluation to evaluate the associated risk for the patient should be recorded as an adverse event and should be investigated and monitored accordingly.

Procedure for notifying SAEs/pregnancy to the sponsor

If an SAE that must be notified to the Clinical Trials Unit occurs, or a pregnancy is recorded, a member of the research team will complete and sign an SAE or pregnancy notification form, which will be sent to the Clinical Trials Unit immediately and always within 24 hours of becoming aware of the event.

The form received will be reviewed and, if applicable, additional information will be requested from the investigator.

When additional Information concerning the SAE/pregnancy is obtained, or it resolves or is unlikely to undergo any change, a follow-up report must also be completed and sent to the Clinical Trials Unit.

If the SAE is suspected to be a SUSAR, the investigator must provide the follow-up information requested by the Clinical Trials Unit.

Any SAE that occurs more than 30 days after the completion of treatment (with no time limit) must be reported if the investigator considers that the SAE is related to the study treatment (in other words, if it is a serious adverse reaction) or if it is medically important.

Expedited reporting of SUSARs to health authorities/CREC

The Clinical Trials Unit is responsible for notifying the AEMPS (clinical trials division of the Sub-Directorate General for Medicines for Human Use), CRECs and autonomous communities of all SUSARs recorded during the study as per the procedure set out in current legislation.

Reporting periods

The maximum period for reporting an individual case of a SUSAR is 15 calendar days from the moment when the sponsor becomes aware of said event. If the SUSAR has led to the death of a patient, or endangered the patient’s life, the sponsor will send the information within a period of 7 calendar days from the moment they become aware of said event. This information will be completed, as far as possible, within the next 8 days.

# 8.- STATISTICS

## 8.1.- Sample Size

The expected number of infusions is 48, distributed into 16 infusions per group. It is recommended to include between 30 and 50 measurements in a pilot study^18,19^.

## 8.2.- Statistical analysis

A general description of all study variables will be performed. Qualitative variables will be expressed as absolute frequencies and percentages, and quantitative variables as the mean and standard deviation (median and 25th and 75th percentiles if they do not follow a normal distribution). The primary endpoint will be evaluated using a chi-squared test (with a Fisher correction if it does not comply with the criteria) and the secondary endpoints will be evaluated by performing a chi-squared test (with a Fisher correction if they do not comply with the criteria) for categorical variables, and Student’s t-test (or Mann–Whitney U-test if they do not follow a normal distribution) will be applied for continuous variables. A Kaplan Meier survival analysis will be applied to determine the time to the infusion-related reaction. A statistical significance level of p<0.05 will be used in all tests. Statistical analysis will be performed using the statistical software packages R (version 3.5.0) and SPSS (version 23.0).

# 9.- DIRECT ACCESS TO SOURCE DATA/DOCUMENTS

The sponsor/Principal Investigator will guarantee that the institution (Araba University Hospital) will allow direct access to the source data or documents for monitoring, auditing, review by the CREC and inspection of the trial by health authorities.

# 10.- ETHICS

The investigator will ensure that this study complies with the principles set out in the Declaration of Helsinki and Good Clinical Practice guidelines. The trial must also comply with ICH GCP guidelines – Addendum (modification) E6 (R2), REGULATION (EU) No 536/2014 OF THE EUROPEAN PARLIAMENT AND OF THE COUNCIL of 16 April 2014 on clinical trials and medicinal products for human use. Royal Decree 1090/2015, of 4 December, regulating clinical drug trials, Drug Research Ethics Committees and the Spanish Clinical Trials Register.

The study report and patient information sheets and informed consent form will be submitted to the Basque Drug Research Ethics Committee for approval.

Patient anonymity and confidentiality will be guaranteed at all times in accordance with the Personal Data Protection Act.

# 11.- FUNDING AND INSURANCE

Inclusion of the study in the Osakidetza insurance policy covering all possible harm that patients may suffer as a result of the research project.

# 12.- PUBLICATION POLICY

The findings of this clinical trial with the study product will be reviewed and discussed by the research team and the sponsor for subsequent publication.

No data obtained during the study will be made available to third parties until an agreement has been reached, in writing, with the sponsor in this regard; data may be made public at conferences or congresses or by publication in a journal.

As an exception to this rule, the researchers may include the title of the trial in their respective CVs. In order to maintain confidentiality, the drug will be identified only by means of its pharmacological group, not by its generic and/or commercial name.

The sponsor/Principal Investigator undertakes to publish the results of this clinical trial, whether positive or negative, preferably in scientific journals, before disclosing them to the non-healthcare audience, irrespective of the obligations to publish the report containing the results in the Spanish Clinical Trials Registry (REec) and the provisions in this respect in (EU) Directive No. 536/2014 of the European Parliament and of the Council of 16 April 2014.
